# Designing informative warning signals: Effects of indicator type, modality, and task demand on recognition speed and accuracy

**DOI:** 10.2478/v10053-008-0064-6

**Published:** 2009-05-04

**Authors:** Catherine J. Stevens, David Brennan, Agnes Petocz, Clare Howell

**Affiliations:** School of Psychology and MARCS Auditory Laboratories, University of Western Sydney

**Keywords:** auditory warnings, workload, modality, icons, semiotics

## Abstract

An experiment investigated the assumption that natural indicators which exploit existing learned associations between a signal and an event make more effective warnings than previously unlearned symbolic indicators. Signal modality (visual, auditory) and task demand (low, high) were also manipulated. Warning effectiveness was indexed by accuracy and reaction time (RT) recorded during training and dual task test phases. Thirty-six participants were trained to recognize 4 natural and 4 symbolic indicators, either visual or auditory, paired with critical incidents from an aviation context. As hypothesized, accuracy was greater and RT was faster in response to natural indicators during the training phase. This pattern of responding was upheld in test phase conditions with respect to accuracy but observed in RT only in test phase conditions involving high demand and the auditory modality. Using the experiment as a specific example, we argue for the importance of considering the cognitive contribution of the user (viz., prior learned associations) in the warning design process. Drawing on semiotics and cognitive psychology, we highlight the indexical nature of so-called *auditory icons* or *natural indicators* and argue that the cogniser is an indispensable element in the tripartite nature of signification.

## Introduction

There is accumulating evidence that natural indicators which not only alert but also inform an operator of a critical situation are recognised with greater accuracy and speed than symbolic warnings. Symbolic auditory warnings that blare, beep, or ring, for example, go unrecognised 40% of the time when there are seven or so different indicators, and require training and retraining to improve retention/detection (Begault, 1994; Momtahan, Hétu, & Tansley, 1993; Patterson, 1982). Recent research has demonstrated some processing advantage for natural indicators where caricatures of everyday sounds alert the operator that there is a problem. By capitalising on an existing learned association between the signal and the event to which it refers, a natural indicator informs the operator of the nature of the problem (Belz, Robinson, & Casali, 1999; Edworthy & Hellier, 2006a, 2006b; Gaver, 1989; Graham, 1999; Keller & Stevens, 2004; McKeown & Isherwood, 2007; Perry, Stevens, Wiggins, & Howell, 2007; Stephan, Smith, Martin, Parker, & McAnally, 2006).

Caricatures of everyday sounds have been considered as auditory icons (Ballas, 1993; Gaver, 1989; Keller & Stevens, 2004). However, as we have argued elsewhere (Petocz, Keller, & Stevens, 2008) the term *icon*, meaning *likeness* or *image*, while having straightforward application in the visual domain cannot be applied in a straightforward manner in the auditory domain. In audition, there are few true auditory icons – that is, where one sound is used to stand for another sound by virtue of resemblance between the two sounds. In the language of semiotics (Peirce, 1932/1960) what has been termed an *auditory icon* is more correctly an index. For clarity, we refer to what have been called *auditory icons* as *natural indicators* where these natural indicators have been adopted or adapted for purposes of conventional indication (Petocz et al., 2008). Abstract warnings where there is no prior systematic relation between signal and event are termed *symbolic indicators* (following Peirce) as their association is determined purely by convention.

 Studies of natural auditory indicators have generally involved a significant amount of training and exposure to the event-signal pairs. In the spirit of human factors and ergonomics, the aim of the present experiment is to expose participants to a defined, relatively short period of training, and to compare recognition of natural indicators and symbolic indicators. Following the recommendations of Patterson (1982), to restrict the set size of symbolic warnings to a maximum of five to eight, we investigated training and recognition of a small set of just four warnings. The modality of warning – visual or auditory – was crossed with warning type. To maximise the ecological validity of the task while presented under controlled lab conditions, task demand was also systematically varied. 

### Advantages of visual icons and auditory natural indicators

Visual icons are used extensively in interface design based on the assumption that visual icons can transcend language barriers and present meaning in a condensed form (Caplin, 2001; Gittens, 1986; McDougall, de Bruijn, & Curry, 2000). Studies of visual icon characteristics – semantic distance, concreteness, familiarity – provide a further explanation of the relative ease of recognition of iconic compared with abstract images. McDougall and colleagues (Isherwood, McDougall, & Curry, 2007; McDougall, Curry, & De Bruijn, 1999, 2001; McDougall et al., 2000) proposed that the importance of visual icon characteristics changes through the time course of experience. For example, semantic distance, operationalised in the present experiment as a natural indicator-conventional indicator comparison, is crucial during initial phases and while visual icon-function relations are learned. Visual icon familiarity is important later, and reflects access to information in long-term memory.

In operational environments where the visual display is often very complex, auditory natural indicators have the potential to be effective warnings because they can be short, are not easily masked by speech or engine noise, are distinct from speech signals, can be used where the visual display is at risk of visual information overload and when the critical event does not make a sound (Keller & Stevens, 2004; Stephan et al., 2006). Reaction times are faster in response to auditory natural indicators compared with tonal and speech warnings (Graham, 1999), and auditory symbolic indicators (Belz et al., 1999; McKeown & Isherwood, 2007).

There are two classes of natural indicators (Petocz et al., 2008). The first involves natural indicators that have been adopted to indicate cause or a correlated object/event. These can include indicators made by humanly manufactured objects (e.g., the sound of a car) but which may nevertheless be considered part of the environment of natural indicators (i.e., the environment into which humans are born). For example, the sound of a car failing to start is correlated with running out of fuel. The second class consists of those natural indicators that have been adapted to exploit naturally occurring shared features (particularly similarity of form or function) between what they naturally indicate and the selected target. For example, the sound of a sink draining (a whirpool form) may be used to signal “tornado” (Keller & Stevens, 2004).

Drawing upon semiotics and psychology, we argued earlier (Petocz et al., 2008) that, because signification is a tripartite relation between signal, referent, and person/cogniser, auditory warning designers cannot afford to neglect the cogniser as an indispensable element. Indeed, the cognitive contribution of the user (viz., prior learned associations) appears to be the most significant factor in determining the effectiveness of warnings.

The cognitive processes involved in recognizing a natural indicator typically include recognition of the source of the sound or image and the activation of long-term memory. Symbolic indicators, on the other hand, need to be learned from the outset; for example, sound bursts with a unique set of frequencies and pause durations that yield a novel and unique pitch and temporal pattern. Features of such a sound or image need to be extracted and become associated with a particular critical event. The association can be strengthened with repeated exposure and directive training. In the context of warning design, both natural and symbolic forms of indication need to be learned but natural indicators have been learned previously and/or exploit some causal, correlational, or similarity of form or function relation. Symbolic indicators, on the other hand, are abstract and learned within an experimental session or within the idiosyncrasies of a particular operational system.

As we have noted (Petocz et al., 2008), it is a truism that connections that have already been (at least partially) learned will be fully learned more easily than connections that have not been previously learned. The purpose of comparing natural and symbolic indicators here is to investigate whether this is the case when the warning set is small (i.e., within the capacity of adult working memory), and in both visual and auditory form.

If warnings are natural indicators for events that they signal, then, during training, accuracy should be greater and reaction time faster in response to natural indicators than to symbolic indicators. This should be the case for warnings presented in either the visual or the auditory modality. We would expect an advantage for natural indicators to hold not just for the learning phase, but also for a test phase in which there are additional and competing demands, as in a dual task.

### Recognition of warnings during high workload

In studies of the effectiveness of different types of warning signals, task demand or workload is rarely investigated. Task demand is a crucial variable for the application and generalisability of results to real-world settings. An assumption, for example, that natural indicators are recognised more often and more quickly than symbolic indicators when task demand is low and the operator is unstressed does not necessarily predict their efficacy during time pressured and/or critical situations. When critical incidents occur there are often many situations including alarms that need attention. To begin to investigate the recognition of natural indicators under more demanding conditions, in the present experiment, participants performed dual tasks – concurrent arithmetic calculations and warning recognition – with a systematic increase in the difficulty of the arithmetic task. Thus we also investigate the effect of task demand on warning recognition speed and accuracy.

### Aim, design, and hypotheses

The aim of the experiment was to investigate the effect of indicator type, modality, and task demand on warning recognition speed and accuracy. The experiment consisted of a 2 x 2 x 2 factorial design: modality (auditory, visual), indicator (natural, symbolic), and task demand (low, high) with repeated measures on the latter two factors. The dependent variables were warning recognition accuracy and reaction time during learning and dual-task test phases. It was hypothesized that (a) in both auditory and visual modalities, natural indicators compared with symbolic indicators elicit greater accuracy and faster reaction time during the learning phase; and (b) in both auditory and visual modalities, natural indicators compared with symbolic indicators elicit greater accuracy and faster reaction time during low and high demand conditions of the dual-task test phase.

## Method

### Participants

Thirty-six adult participants (32 females and 4 males) from the University of Western Sydney took part in the study for which they received course credit. The mean age of the sample was 20.72 years, (*SD* = 4.62, range 18-42 years). Eighteen participants were presented with auditory signals and 18 with visual signals. During the test phase, within each modality, 9 participants completed the low demand dual task first followed by high demand, while the remaining 9 participants completed the high demand dual task first followed by low demand. Also during the test phase, within each modality, 9 participants were presented initially with blocks of natural indicators followed by blocks of symbolic indicators, and 9 participants completed symbolic indicator blocks before natural indicator blocks. All participants had normal hearing and normal or corrected-to-normal vision.

### Stimuli

The auditory and visual natural indicators used in the experiment were rated as a set of seven in a separate stimulus selection task (*N* = 40; mean age: 22 years) as being highly related (means of 3.77-4.33 out of 5) with specified critical events. Ratings of association between symbolic indicators and events with which they were arbitrarily paired were low with a mean association rating of 1.56 (*SD* = 0.72) on a scale from *totally unrelated* (1) to *highly related* (5). Four critical aviation events that could potentially lead to an accident were selected. Symbolic auditory and visual indicators, and natural auditory and visual indicators were designed for each of these.

The critical events were presented as “clickable” buttons on a computer screen, equidistant from one another. Warnings were presented either visually at the top of the computer screen for 1000 ms or auditorily through headphones, also lasting for 1000 ms.

A mathematical addition task, presented visually and concurrently on an adjacent computer, was constructed in the form of low and high demand conditions. The low demand version consisted of three numbers, all less than five (e.g., 1 + 2 + 3), presented in the middle of the computer screen. The high demand task consisted of two double figure numbers (e.g., 26 + 49). Participants were required to mentally add the numbers together and then say the answer aloud. The addition task was displayed on the screen for 2000 ms. Both low and high demand conditions consisted of a total of 16 additions. Warnings were presented intermittently with approximately four addition tasks presented to the occurrence of one warning.

#### Auditory Natural Indicators

The auditory natural indicators were obtained from the websites www.sounddogs.com and www.findsounds.com. The four natural indicators were from the first class, that is, adopted to indicate cause or a correlated object/event. For example, the sound of coughing is correlated with an excess of a dangerous gas such as carbon monoxide. All sampled everyday sounds were 1 s in duration, 16-bit mono, and standardized to a sample rate of 44.1 kHz, with normalized amplitude. Descriptions are given in Table 1.

**Table 1.** Visual and Auditory Natural Indicators Used as Stimuli

Critical event	Visual natural indicator description	Visual natural indicator	Auditory natural indicator description
Low fuel	Petrol pump	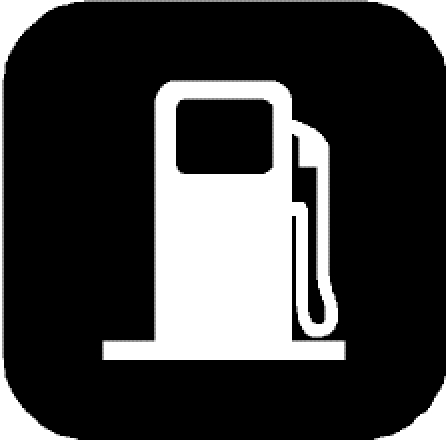	Car failing to start
Carbon monoxide	Skull and crossbones	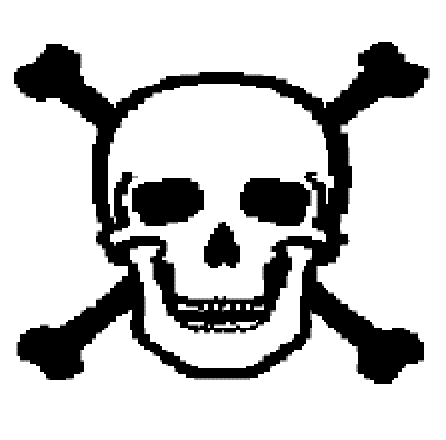	Coughing
Ground proximity	Plane diving into mountain	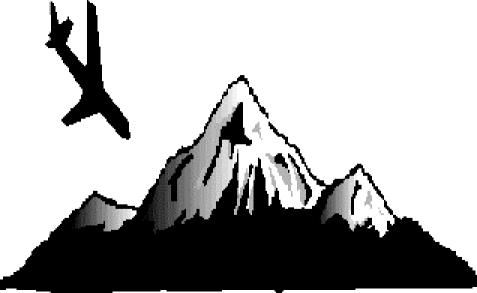	Explosion
Engine fire	Fire extenguisher	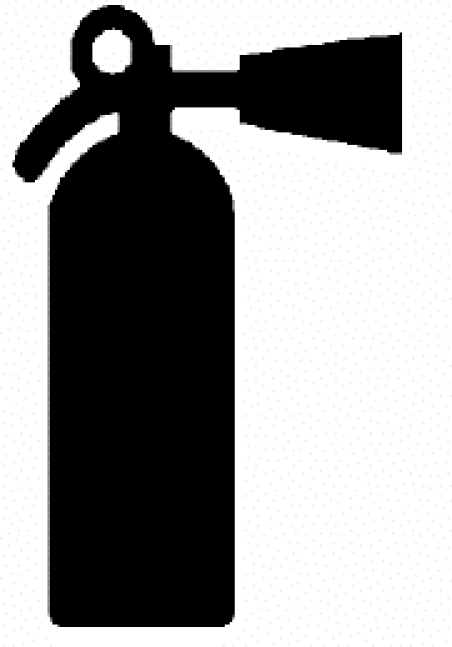	Fire engine siren

#### Auditory Symbolic Indicators

 The auditory symbolic indicators consisted of tones with normalized amplitude, 44.1 kHz sample rate, 16-bit resolution, mono, and were all 1 s in duration. The sounds were designed based on guidelines set by Patterson (1982) in which a burst of sound is first created and repeatedly played over the duration of the signal. Each sound burst had its own set of frequencies and pause durations, giving each warning a unique pitch and temporal pattern. The duration of the bursts varied from 0.19 s to 1 s. The tones and upper harmonics of periodic sounds were selected from frequencies in the range 150–3000 Hz. The auditory symbolic indicators stood in no obvious relation to the events with which they were paired. 

#### Visual Natural Indicators

The visual natural indicators were obtained from the website www.clipart.com and are depicted in Table 1. The visual natural indicators were designed to be similar to their auditory counterparts in the way that they related to their targets via causal/correlational based indication. For example, the image of a petrol pump was not used to indicate “petrol pump”, which would have been a purely icon-based relation; instead, it was used to indicate something associated with a petrol pump (low fuel). All clipart images were shown in black and white.

#### Visual Symbolic Indicators

To help to ensure that image complexity was comparable across the set of visual natural and symbolic indicators, the visual symbolic indicators were obtained by enlarging a small section of visual images from clipart, other than those being used as visual natural indicators. All symbolic indicators were selected on the basis that there was no obvious relationship between the indicator and the event with which it was paired. All images were shown in black and white. Visual symbolic indicators for each of the four events are shown in Table 2.

**Table 2.** Visual Symbolic Indicators Used as Stimuli

Critical event	Visual symbolic indicator
Low fuel	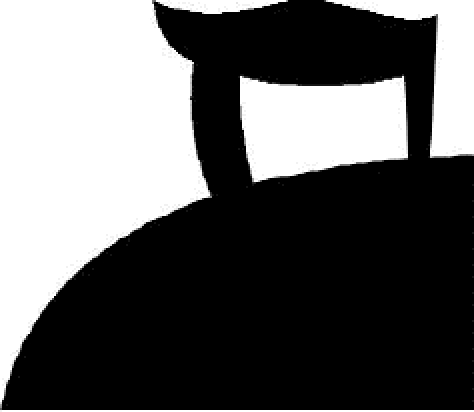
Carbon monoxide	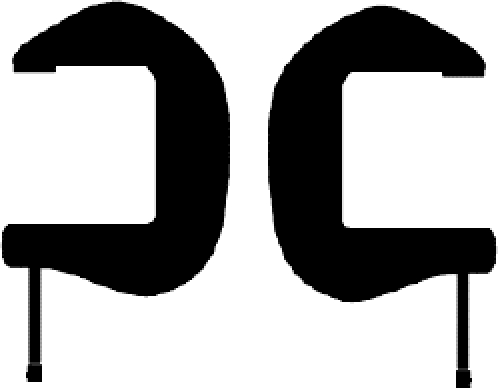
Ground proximity	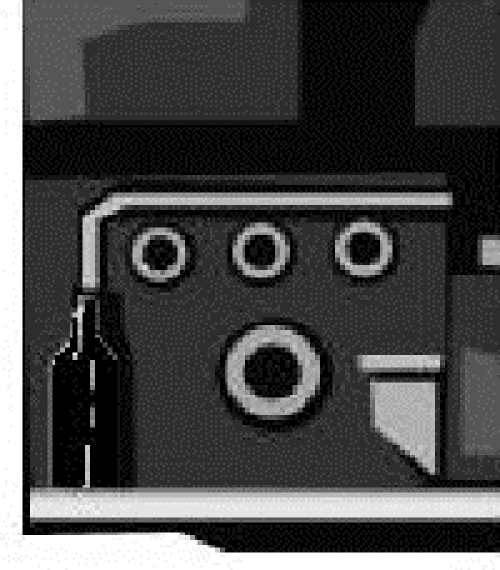
Engine fire	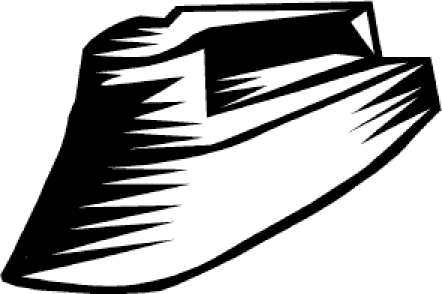

### Equipment

The experiment was programmed in PowerLaboratory version 1.0.3 (Chute & Westall, 1996), and presented to participants on a Macintosh iBook G4. The concurrent addition task was programmed in SuperLab Pro 1.74 and presented to participants on a Macintosh Power Book G4. Auditory warnings were played through Koss stereo headphones.

### Procedure

The procedure was approved by the University of Western Sydney Human Research Ethics Committee. An information sheet was distributed and participants provided written consent before the experiment began. Participants were randomly assigned to either the visual or the auditory condition and were provided with some context for each of the warnings by reading a Critical Aviation Events Information sheet. For example, “Carbon monoxide is a colorless, odorless gas that can be produced through the burning of fossil fuels. If sufficient levels of carbon monoxide enter the cockpit, the pilot can be rendered unconscious”. Participants were randomly assigned to the indicators (symbolic or natural) that they would be exposed to and learn first. They were trained on the relation between each of the warnings and the corresponding event, then tested on the warning-event relation, until they had had a total of 16 presentations. Corrective feedback was given after each response. This design contrasts with previous studies wherein participants have been trained to a certain criterion level of performance, for example (Keller & Stevens, 2004; Stephan et al., 2006; see also Gaver, Smith, & O’Shea, 1991). We adopt a human factors approach involving minimal training and a stimulus set that should not exceed the capacity of adult working memory.

In the test phase, participants were required to perform a visual addition task in which numbers appeared briefly on the screen and they were required to add the numbers together as fast and as accurately as possible, while still responding to the warnings when they were presented. The visual addition task was presented through an adjacent computer, requiring that participants divide their attention between two computers. The experimenter recorded participants’ mental addition vocal responses manually. A second phase of the experiment (comprising both learning and test phases) involved the same procedure using a new set of signals from the warning type not yet tested. The experiment took 25-30 min.

## Results

Descriptive statistics relating to arithmetic accuracy in the test phase are shown in Table 3. Warning recognition accuracy and reaction times on correct responses in learning and test phases are displayed in Tables 4. and 5, respectively.

As a manipulation check, performance on the high and low demand versions of the concurrent arithmetic task was computed. There was a main effect of task demand, *F*(1, 34) = 160.4, *p* < .05, Cohen’s *d* = 2.30, with a significant difference between arithmetic scores in the high demand (*M* = 6.69, *SD* = 4.47) and low demand (*M* = 14.43, *SD* = 1.64) conditions. There was no main effect of modality or indicator on arithmetic scores and no interactions between modality, indicator, or task demand factors.

**Table 3.** Mean Accuracy on the Arithmetic Task in the Dual Task Test Phase (max. = 16)

		Low demand		High demand	
Indicator	Modality	*M*	*SD*	*M*	*SD*
Natural	Auditory	13.61	2.12	5.83	4.84
	Visual	14.83	1.10	7.50	4.50
	Total	14.22	1.77	6.67	4.68
Symbolic	Auditory	14.39	1.85	6.33	4.42
	Visual	14.89	1.08	7.11	4.19
	Total	14.64	1.51	6.72	4.26

**Table 4.** Mean Warning Recognition Accuracy in the Learning Phase (max. = 16) and Low and High Demand Test Phases (max. = 4)

		Learning phase		Low demand		High demand	
Indicator	Modality	*M*	*SD*	*M*	*SD*	*M*	*SD*
Natural	Auditory	14.83	1.47	3.44	0.86	3.67	0.59
	Visual	15.67	0.59	3.44	0.70	3.33	0.97
	Total	15.25	1.18	3.44	0.77	3.50	0.81
Symbolic	Auditory	10.56	2.94	2.72	1.23	2.33	1.08
	Visual	11.67	4.51	2.33	1.03	2.72	1.02
	Total	11.11	3.79	2.53	1.13	2.53	1.06

**Table 5.** Mean Warning Recognition Reaction Times in the Learning Phase and Low and High Demand Test Phases (correct responses only)

		Learning phase		Low demand		High demand	
Indicator	Modality	*M*	*SD*	*M*	*SD*	*M*	*SD*
Natural	Auditory	4209	343.3	2670	645.6	2092	316.0
	Visual	3990	405.4	2163	661.8	2195	708.6
	Total	4099	386.5	2431	699.5	2143	543.3
Symbolic	Auditory	4671	674.1	2442	651.1	2778	997.0
	Visual	4323	688.9	2023	637.7	2348	671.2
	Total	4497	694.5	2232	669.7	2563	865.4

Note. Reaction times were measured in milliseconds, from stimulus onset.

### Learning Phase

It was hypothesized that natural indicators compared with symbolic indicators elicit greater accuracy during the learning phase. Significantly greater accuracy was recorded during the learning phase in response to natural indicators than to symbolic indicators, *F*(1, 34) = 44.6, *p* < .05, Cohen’s *d* = 1.47. There was no main effect of modality (Auditory *M* = 12.69, *SD* = 2.21; Visual *M* = 13.67, *SD* = 2.55) and no modality x indicator interaction.

It was hypothesized that natural indicators compared with symbolic indicators elicit faster reaction time (RT) during the learning phase. RTs of correct responses in the learning phase were significantly faster in response to natural indicators than to symbolic indicators, *F*(1, 34) = 13.6, *p* < .05, Cohen’s *d* = 0.71. There was no main effect of modality (Auditory *M* = 4439.67, *SD* = 508.70; Visual *M* = 4156.16, *SD* = 547.12) and no modality x indicator interaction.

### Test Phase

The second hypothesis was that, in both visual and auditory modalities, natural indicators compared with symbolic indicators elicit greater accuracy and faster reaction times during high and low demand conditions of the dual-task test phase.

With respect to recognition accuracy, there was a main effect of indicator, *F*(1, 34) = 25.3, *p* < .05, Cohen’s *d* = 0.99, and a significant interaction between modality, indicator, and task demand, *F*(1, 34) = 8.1, *p* < .05. In the high demand condition, accuracy was significantly greater in response to natural indicators than to symbolic indicators in both the auditory modality, *F*(1, 17) = 22.7, *p* < .05, Cohen’s *d* = 1.53, and visual modality, *F*(1, 17) = 5.6, *p* < .05, Cohen’s *d* = 0.61. Similarly, in the low demand condition, accuracy was significantly greater in response to natural indicators than to symbolic indicators in both the auditory, *F*(1, 17) = 4.8, *p* < .05, *d* = 0.68, and visual modalities, *F*(1, 17) = 11.9, *p* < .05, *d* = 1.18.

With respect to reaction times, there were two significant interactions: modality x task demand, *F*(1, 34) = 4.6, *p* < .05, and indicator x task demand, *F*(1, 34) = 9.8, *p* < .05. In the high demand condition, RTs were significantly faster in response to natural indicators than to symbolic indicators in the auditory modality, *F*(1, 17) = 8.7, *p* < .05, Cohen’s *d* = 0.93, but there was no significant difference between natural and symbolic indicator RTs in the visual modality. Contrary to the hypothesis, there was no significant difference between natural and symbolic indicators with respect to RT in the low demand, auditory modality condition or the low demand, visual modality condition.

## Discussion

This experiment investigated the effect of indicator type, modality of presentation, and task demand on warning recognition speed and accuracy during training and dual task test phases. As hypothesized, during the learning phase, natural indicators (or caricatures of everyday sounds and objects) relative to symbolic indicators elicited greater recognition and faster RTs. This pattern of responding was upheld in test phase conditions with respect to accuracy. The pattern was observed in RT only in the test phase involving high demand and the auditory modality.

Results from the learning phase provide support for the general hypothesis that in learning signal-event relations there is an advantage for those natural indicators that have been previously learned and are now being exploited for indication (Petocz et al., 2008). This pattern occurs even when a set of just four warnings is used, corroborating findings of others (Stephan et al., 2006; see also Belz et al., 1999; Gaver et al., 1991; McKeown & Isherwood, 2007; Perry, Stevens, Wiggins, & Howell, 2007). Natural indicators elicit recognition of the source of the sound or referent of the image, and activate associations in long-term memory. Symbolic signal-event relations need to be learned within an operational context. While RTs in the present experiment were relatively slow in response to warnings during training, they improved in the dual-task test phase.

The expected pattern of results has been obtained in accuracy but not fully in RT scores of the test phase. Natural indicators elicited significantly faster RTs in the high demand task with warnings in the auditory modality but not in the visual modality and not in the low demand condition. One explanation derives from scrutiny of the concurrent task arithmetic scores and an apparent weighting of tasks by participants. The mean arithmetic score in the low demand condition was approximately 14 out of 16 whereas in the high demand condition the maximum mean was around 7 out of 16. Participants performed well on simple addition tasks to the detriment of speed in recognizing auditory natural indicators. That this occurred only in the auditory modality may be attributable to the greater degree of possible ambiguity in the auditory natural stimuli. For example, the sound of coughing was used as a natural auditory indicator for carbon monoxide. However, it is a natural indicator of many other situations such as a person with a cold, a person choking, a person smoking heavily or (significantly for the present context) that there is a(n engine) fire. The same can be said for the sound of a car failing to start and the sound of an explosion. Of the four auditory natural indicators used in the experiment, the fire engine siren is perhaps the only one that is as closely connected to its target and as unconfounded with other possible connections as is its visual counterpart (the image of a fire extinguisher). In contrast, the visual natural indicator of a petrol pump is a typical fuel indicator in motor vehicles, and the skull and crossbones are a familiar indicator of poison. Visual natural indicators thus may already be better learned at the start of the experiment than are auditory natural indicators, and may also be more distinctive in the sense of being less liable to confounding with other prior learned associations. The visual advantage was observed despite the fact that participants were required to attend to two monitors in the visual group while participants in the auditory group viewed only one monitor. Of course, there are several other factors which may have contributed to the visual advantage (e.g., temporal differences in stimulus registration, auditory interference from the requirement to state aloud the solution of the mathematical problem). However, it is clear from other research (Petocz et al., 2008) that, in general, the cognitive contribution (viz., prior learning) of participants and users must also be considered in warning design.

In the high demand condition, participants performed poorly on the mental arithmetic, possibly guessing, and leaving more cognitive resources for relatively fast warning recognition. The symbolic auditory indicators under low demand elicited more optimal performance with good arithmetic accuracy and, relative to the natural indicators, faster RTs. However, under conditions of high demand, arithmetic was again poor and auditory symbolic indicators were recognized slowly. The use of a demanding concurrent task has brought into relief the potentially complex and operationally important interaction between task load and indicator type.

A further advantage of the auditory modality may manifest when coupled with a visually-presented arithmetic task. However, the present results do not suggest interference from visually-presented indicators when the learning phase visual versus auditory modality accuracy scores are compared with test phase visual versus auditory modality scores. Similarly, in arithmetic scores there was no significant effect of modality.

 The present experiment was designed specifically to contrast the design used by Perry et al. (2007) and, in the spirit of a human factors approach, to keep training to a minimum. Thus, rather than training all participants to 100% criterion level of performance as we have done in the past, all participants were exposed to a set number of training trials. The present results suggest that training to a criterion level of performance may be important especially in the case of symbolic indicators. 

While there was no main effect of task demand in the test phase, demand did interact with modality in both indicator recognition and RT scores. This provides evidence of the need to examine the setting in which warnings will be used not only from the perspective of ambient noise, potential maskers, and the complexity of existing auditory and visual displays, but also the nature of the operational task and the load that it incurs. There is need also for effects of demand and indicator to be investigated in settings that approach the operational context such as an Advanced Aviation Training Device (AATD).

The importance of context in warning design is underscored by the present results. The artificial environment is always embedded, both physically and psychologically, in the natural environment that includes the learned associations of the user. Not only is it a truism that an already-learned association will be more easily and quickly learned than one which has not yet been learned, but it is also true that if the referent is just one of a large set of equally salient associations, such that learning the connection requires the learner to “unlearn” or ignore those other meanings, then a natural indicator may actually be *less* effective than a newly designed symbolic indicator that is free of “excess baggage”. Thus, while exploiting natural indicators is a good idea, there are advantages and disadvantages. On the other hand, the user is likely to bring some association even to symbolic connections. This is confirmed by the fact that, in the present experiment, ratings of symbolic associations were typically higher than zero.

These observations suggest that the cognitive contribution of the user is not just one among many equally salient and cumulatively contributing factors to be taken into account for the design of warnings. Instead, the cognitive contribution of the user infiltrates other factors which are often treated independently as in perceived stimulus complexity, meaning, semantic distance, perceived aesthetic appeal, and so on.
